# Long-term follow-up MRI shows no hastening of adjacent segment degeneration following cervical disc arthroplasty

**DOI:** 10.1038/s41598-022-17652-8

**Published:** 2022-08-03

**Authors:** Benedikt W. Burkhardt, Lukas Baumann, Andreas Simgen, Gudrun Wagenpfeil, Philipp Hendrix, Wolfgang Reith, Joachim M. Oertel

**Affiliations:** 1 Department of Neurosurgery, Saarland University Medical Center and Saarland University Faculty of Medicine, Homburg-Saar, Germany; 2 Department of Neuroradiology, Saarland University Medical Center and Saarland University Faculty of Medicine, Homburg-Saar, Germany; 3 Institute for Medical Biometry, Epidemiology and Medical Informatics (IMBEI), Saarland University Faculty of Medicine, Homburg-Saar, Germany; 4 Klinik für Neurochirurgie, Universitätsklinikum des Saarlandes und Medizinische Fakultät der Universität des Saarlandes, Kirrbergerstrasse 100, Gebäude 90.5, 66421 Homburg-Saar, Germany

**Keywords:** Prognosis, Quality of life, Outcomes research

## Abstract

Cervical disc arthroplasty is an established procedure, but studies with data on long-term clinical outcome, reoperation for symptomatic adjacent segment degeneration (sASD), and degenerative changes based on MRI findings are rare. Thus, a file review was performed and patients with complete documentation of neurological status at preoperative, postoperative, 12 month, 3–4 years follow-up including surgical reports for reoperation with a minimum follow-up of 9 years were included. Final follow-up assessment included a physical examination, assessment of pain levels, Odoms criteria, Neck disability index. The degeneration of each cervical segment at preoperative and at final follow-up was assessed using an MRI. Forty-six out of 68 included patients participated, the mean follow-up was 11 (range 9–15) years, at which 71.7% of patients were free of arm pain, 52.2% of patients were free of neck pain, 63% of patients had no sensory dysfunction, and full motor strength was noted in 95.6% of patients. The clinical success rate was 76.1%, the mean NDI was 12%. Overall repeated procedure rate was 17%, the reoperation rate for sASD was 9%, and removal of CDA was performed in 4%. MRI showed progressive degeneration but no significant changes of SDI from preoperative to final follow-up.

## Introduction

Degenerative disorders of the cervical spine are a common cause for neck pain, radiculopathy and myelopathy^1^. Since its first introduction in the 1950s, the anterior cervical discectomy and fusion (ACDF) procedure evolved to increase fusion and restore lordosis^2–5^. Long term clinical outcome demonstrated good results up to more than 20 years of follow-up^6–9^. However, fusion reduces segmental motion which is believed to be the cause for increased stress at the segments adjacent to the fusion and to hasten degeneration^10^. The term “adjacent-segment degeneration” (ASD) was defined later on and repeated procedure for symptomatic ASD (sASD) is an adverse event following ACDF^11–13^. Cervical disc arthroplasty (CDA) was developed to preserve motion of the diseased segment following decompression, reduce ASD and mitigate the rate of reoperation for sASD. There is a limited number of long term studies and conflicting results regarding the rate for reoperation due to sASD following CDA and ACDF have been reported^14–16^.

The present study reports long-term (more than 9 years) clinical data following CDA and evaluates the segmental degeneration using MRI criteria following CDA.

## Material and methods

An institutional database was reviewed to identify all consecutive cases of cervical disc arthroplasty (CDA) for the treatment of degenerative disorders. To obtain long-term clinical outcome data and MRI data only charts of the years from 01/2004 to 12/2011 were reviewed.

All cases matching the following criteria were included for further evaluation: complete preoperative and postoperative neurological status during hospitalization, complete preoperative MRI of the cervical spine, no previous cervical spine surgery, a precise surgical report of the procedure, outpatient visit documentation at a minimum of 12 and 36 months postoperatively, accurate documentation in case of repeated cervical spine procedure, and complete contact information.

Patients who did not fulfil all of the aforementioned criteria were excluded for evaluation. All patients who fulfilled the aforementioned inclusion criteria were subsequently contacted for final follow-up evaluation.

### Ethical approval

The study design was approved by local ethics committee (“Ethikkommission der Ärztekammer des Saarlandes” reference number: 150/17) and informed consent was obtained from all the participants involved in this study. The study was performed in accordance to the Declaration of Helsinki.

### Assessment of clinical outcome based on the patients file

Retrospectively, each patient’s file was reviewed thoroughly to assess the preoperative, postoperative, 12 month, and minimum of 36 month follow-up neurological status. Special focus was pointed out on symptoms such as muscle strength, sensory deficits, the presence of arm and neck pain, gait disturbance, dysphagia and hoarseness. Muscle weakness was assessed according to the grading by Janda.10.

The intensity of arm and neck pain was defined as follows: none was considered 0 points, mild pain was considered 1 to 2 points, moderate pain was considered 3 to 5 points, severe pain was considered 6 to 8 points, and extreme pain was considered 9 to 10 points.

### Final follow-up evaluation

All patients who participated for final follow-up evaluation underwent a physical examination and a standardized questionnaire including the EQ-5D questionnaire, Neck Disability Index (NDI), Odom’s criteria, and questions regarding the intake of pain medication.

Excellent and good clinical outcome according to Odom`s criteria were considered as clinical success. In addition, a MRI scan of the cervical spine was performed to evaluate the grade of degeneration of the cervical spine as described below.

### MRI evaluation and assessment parameters

**Figure 1:**
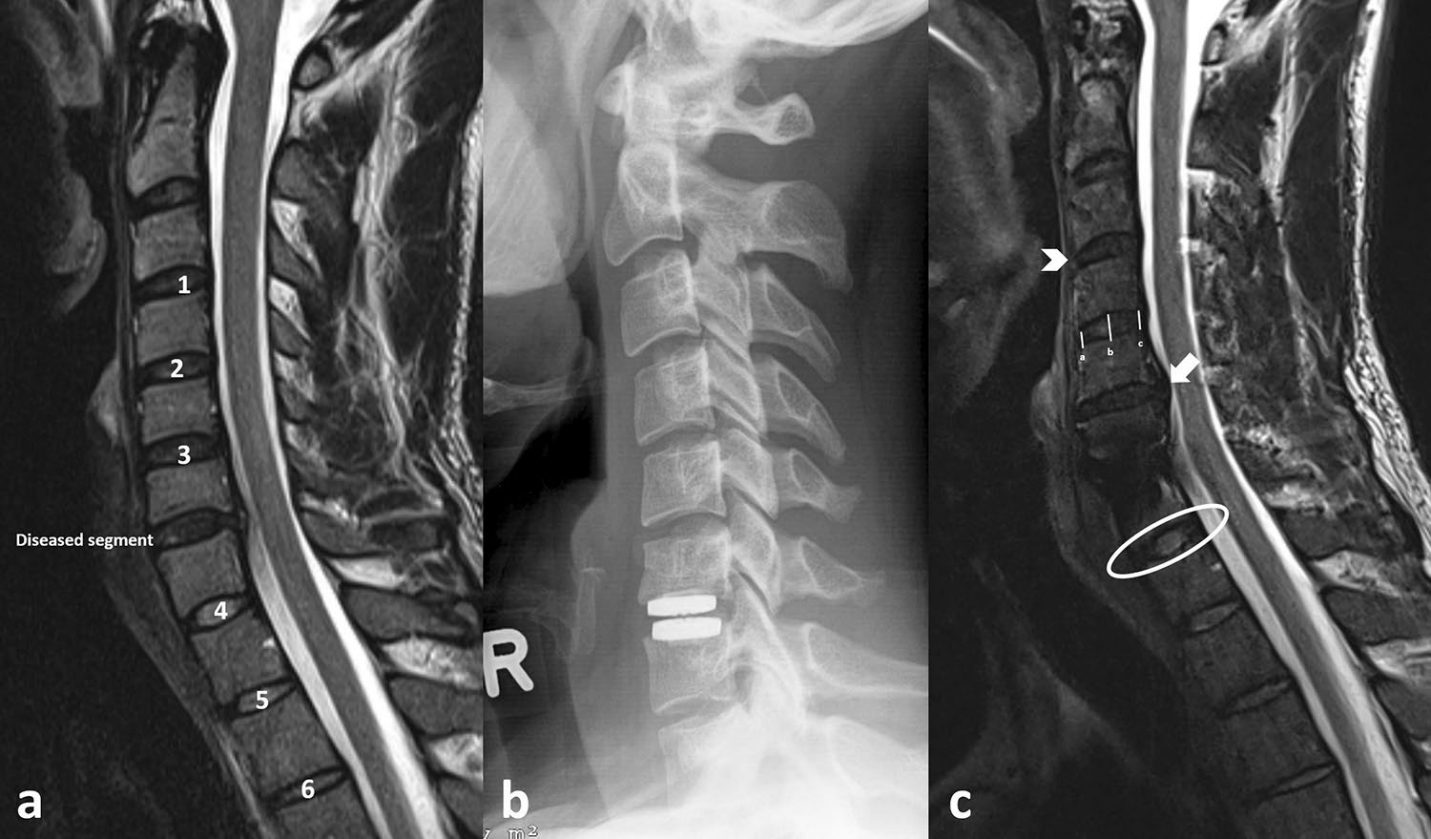
MRI evaluation and SDI measurements. (**a**) Preoperative MRI scan, 1 = Distal cranial adjoining segment, 2 = Proximal cranial adjoining segment, 3 = Cranial adjacent segment, 4 = Caudal adjacent segment, 5 = Proximal caudal adjoining segment, 6 = Distal caudal adjoining segment (**b**) Postoperative radiograph after CDA implantation. (**c**) Follow-up MRI scan, white arrow head = no anterior disc protrusion (0 point), a = anterior disc height, b = middle disc height, c = posterior disc height, white arrow = disc material protruding behind the margin of the vertebral body (1 point), white ring = disc signal intensity (Bright as or slightly less bright than CSF—0 point).

The preoperative and the final follow-up MRI scan were analysed by two blinded reviewers. Each reviewer assessed the cranial and caudal segment adjacent to the CDA side. Also, if anatomical feasible the proximal and distal cranial adjoining segments as well as the proximal and distal caudal adjoining segments were assessed. Therefore, a total of six segments were assessed in case of a CDA at the C5/6 segment. The number assessable segments which are located cranial to the CDA, decreases in case of a CDA procedure at an upper cervical segment (i.e. C3/4 and C4/5), see Fig. 1.

**Figure 2:**
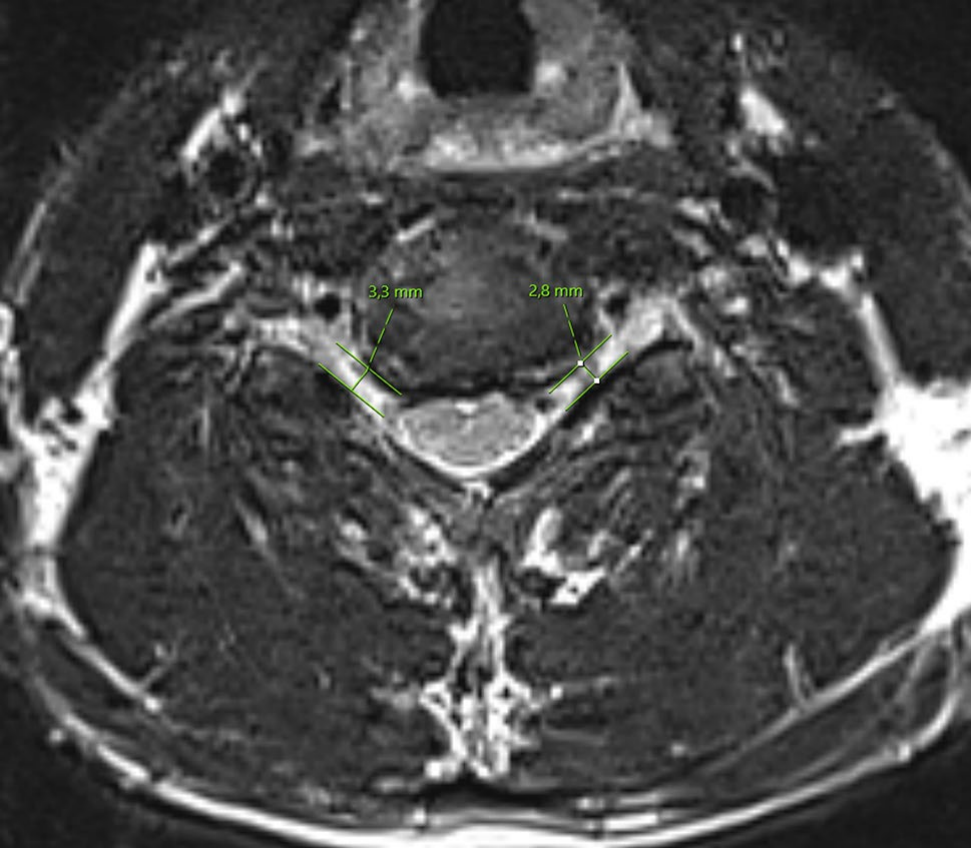
SDI measurement. Bilateral foraminal stenosis (1 point).

The segmental degeneration index (SDI), which is a five category grading was used for the assessment of segmental degeneration (see Table 1 and Figs. 1 and 2)^17–20^. A detailed account to the MRI protocol and evaluation process has been reported previously^18^.

**Table 1:** Five category grading system for segmental degeneration.

Category	Grade of degeneration	Points
Disc signal intensity	Bright as or slightly less bright than cerebrospinal fluid	0
	Dark and/or speckled	1
	Almost black	2
Posterior disc protrusion	Disc material confined within the posterior margin of the vertebral body	0
	Disc material protruding beyond the posterior margin of the vertebral body without compression	1
	Beyond the vertebral body with cord compression	2
Anterior disc protrusion	Disc material confined within the anterior margin of the vertebral body	0
	Disc material protruding beyond the anterior margin of the vertebral body	1
Narrowing of disc space*	0–25% difference of disc height narrowing between the adjacent segment compared to the median disc height of non-adjacent segment	0
	25–50% difference of disc height narrowing between the adjacent segment compared to the median disc height of non-adjacent segment	1
	> 50% difference of disc height narrowing between the adjacent segment compared to the median disc height of the non-adjacent segment	2
Foraminal stenosis	Axial foraminal diameter > 4.0 mm	0
	Axial foraminal diameter < 4.0 mm	1

*The disc height was calculated as the mean value of the anterior, the middle and the posterior DH of each segment (see Fig. 1).

Each of the five categories is rated with 0–2 points depending on the degree of degeneration. The total number of possible points varies from 0 to 8. Once a category could not be evaluated with absolute certainty it is excluded from assessment. The maximum of possible points was downgraded according to this specific category. For each segment the total of assessed points is then divided by the maximum of possible points.

Therefore, the SDI might range from 0.0 to 1.0. The SDI increases as the degree of degeneration increases. A SDI score of less than 0.333 as defined as mild, a score of 0.334 to 0.667 was defined as moderate and a score of more than 0.667 was defined as severe.

### Statistical analysis

SPSS software version 25 was used for statistical analysis (IBM, Armonk, NY, USA). A two sided t-test was used to compare the grade of degeneration according to the preoperative and follow-up SDI scores. A linear regression analysis was used to assess the influence on gender, number of operated segments, and the type of disc prosthesis on the grade of degeneration.

Agreement between the reviewers and the intra class correlation (ICC) were assessed. An ICC of more than 0.8 is considered to indicate very good interrater reliability.

## Results

### Patient demographics and CDA procedure related findings

A total of 68 patients fulfilled the inclusion criteria and 46 (67.6%) patients (23 males and 23 female) agreed to participate for a final follow-up assessment and MRI scan of the cervical spine. The mean age at initial CDA procedure was 45.6 years (range: 30–63 years). Eighteen (39.2%) patients had a history of smoking prior to CDA procedure.

In 42 patients the indication for surgery was acute onset of cervical radiculopathy due to cerivical disc herniation without improvement of symptoms after at least 6 weeks of conservative therapy. In cases with acute onset of motor weakness $\le$ 3/5 CDA procedure was performed without completion of 6 weeks of conservative therapy. In four cases cervical stenosis and spondylosis with signs of myelopathy were noted prior to CDA procedure.

Each procedure was performed by two neurosurgeons including a senior consultant neurosurgeon with at least 10 years of experience in spine surgery and a neurosurgical resident.

One-level CDA was performed in 35 and two-level CDA in 11 cases. One-level CDA procedures was performed at the level of C3/4, C4/5, C5/6, C6/7 and C7/T1 in two, three, 15, 14, and one cases, respectively. Two-level CDA procedure of C4/5 and C5/6, and C5/6 and C6/7, in five cases each respectively. In one case a two-level CDA was performed at C4/5 and C6/7. In all cases of two-level CDA both implants were of the same model and flexibility. A compilation of patient characteristics and operated segments is shown on Table 2.

**Table 2:** Patient characteristics and operated segments.

Patient Characteristics and operated segments		
Gender		**100%**
male	23	50%
female	23	50%
Mean age in years		45.6
Smoking status positiv preoperativ	18	39.2%
Diagnosis (total)	46	100%
Disc herniation	42	91.3%
Spondylosis with myelopathy	4	8.7%
Single-level fusion (total)	35	76.1%
C3/4	2	4.3%
C4/5	3	6.5%
C5/6	15	32.6%
C6/7	14	30.4%
Two-level fusion (total)	11	23.9%
C4/5 and C5/6	5	10.8%
C5/6 and C6/7	5	10.8%
C4/5 and C6/7	1	2.1%

### Clinical finding

#### Preoperatively

Radicular arm pain was noted in 35 (76.1%) of patients with a mean pain intensity of 6.2 (range: 2–10). Neck pain was noted in 35 (76.1%) of patients with a mean intensity of 3.9 (range: 1–10). A motor weakness was noted in 12 (26.1%), a sensory deficit in 31 (67.4%) patients, and a gait disturbance was noted in 3 (6.5%) patients.

#### Intraoperatively

No implant associated complication was noted intraoperatively according to the surgical reports. In twenty-four patients a M6 prosthesis (Spinal Kinetics, Sunnyvale, CA, USA), in thirteen patients a ProDisc-C prosthesis (Synthes GmbH, Oberdorf, Switzerland), and in nine patients an active-C prosthesis (Aesculap AG, Tuttlingen, Germany) was implanted at CDA procedure.

#### Postoperatively

An improvement of arm pain was noted in 43 (93.7%) patients, whereas 3 (6.3%) patients had increased arm pain. Improvement of neck discomfort and neck pain was noted in 45 (97.8%) patients, whereas 1 (2.2%) patient had increased neck pain. None of the patients had worsening or new onset of paresis. In 16 (84.2%) patients regained full muscle strength during hospitalization, and 29 (58.0%) patients reported to be free of sensory disturbance. One (2.1%) patient each developed a new sensory deficit, temporary urinary retention, hoarseness, and eight (17.4%) patients reported dysphagia.

#### Final follow-up evaluation

The mean final follow-up was 11 years (range: 9–15 years) at which 46 (23 males and 23 female) patients participated. The mean age at final follow-up was 56 years (range: 40–72 years).

Twenty-four (52.2%) patients denied any kind neck pain or discomfort. The mean neck pain intensity was 2.5 (range 0–10) on the NRS. The mean NDI was 12% (range: 0–54%). Fifteen (32.6%) patients reported occipital headache.

Thirty-three (71.7%) patients had no arm pain or discomfort. The mean arm pain intensity was 1.3 (range 0–9) on the NRS.

Twenty-nine (63.0%) patients reported no sensory dysfunction. Ten (83.3%) out of 12 patients regained full motor strength, and a mild 4 +/5 paresis was noted in two patients. A gait disturbance was noted in four (8.6%) patients.

A compilation of clinical data at 12 months` follow-up, 3 to 4 years` follow-up, and final follow-up with respect to arm- and neck pain, motor weakness, sensory deficits and gait disturbance presented on Table 3.

**Table 3:** Compilation of clinical outcome.

	Preoperative	12 months´ follow-up	3–4 years´ follow-up	Final follow-up (9–15 years)
	**Neck pain**	**Neck pain**	**Neck pain**	**Neck pain**
None (NRS: 0)	23.5%	51.4%	46.9%	52.2%
Mild (NRS:1–2)	29.4%	38.2%	42.8%	8.7%
Moderate (NRS: 3–5)	38.2%	10.3%	8.2%	13.0%
Severe (NRS: 6–8)	5.8%	0%	2.0%	21.7%
Extreme (NRS 9–10)	2.9%	0%	0%	4.3%
Occipital headache	None	None	6.1%	32.6%
	**Arm pain**	**Arm pain**	**Arm pain**	**Arm pain**
None (NRS: 0)	23.9%	79.4%	70.1%	71.7%
Mild (NRS:1–2)	14.7%	16.2%	15.6%	2.2%
Moderate (NRS: 3–5)	32.4%	2.9%	10.2%	4.3%
Severe (NRS: 6–8)	22.1%	1.5%	4.0%	19.5%
Extreme (NRS 9–10)	7.3%	0%	0%	2.2%
	**Sensory deficit**	**Sensory deficit**	**Sensory deficit**	**Sensory deficit**
None	32.6%	58.7%	69.4%	63.0%
Existing	67.4%	41.3%	30.6%	37.0%
*Unchanged*		*2.2%*	*8.2%*	30.4%
*Improved*		*28.2%*	*22.4%*	4.3%
*New onset*		*10.8%*	*n.a*	2.1%
*Worsening*				
	**Motor weakness**	**Motor weakness**	**Motor weakness**	**Motor weakness**
None	73.9%	95.7%	95.7%	95.3%
Existing	26.1%	4.3%	4.3%	4.3%
*4/5*	*15.2%*	*4.3%*	*4.3%*	*4.3.%*
*3/5*	*6.5%*	*n.a*	*n.a*	*n.a*
≤ *2/5*	*4.3%*	*n.a*	*n.a*	*n.a*
	**Gait disturbance**	**Gait disturbance**	**Gait disturbance**	**Gait disturbance**
None	95.5%	100%	100%	91.4%
Existing	6.5%	0%	0%	8.6%

.

Head rotation was without any limitations in 24 (52.2%), with slight limitations in 13 (28.3%), and painful in 9 (19.6%) patients. Lateral bending of the cervical spine was without limitations in 20 (43.5%), with slight limitations in 14 (30.4%), and painful in 12 (26.6%) patients. Reclination was without limitations in 39 (84.8%), limited in one (2.2%), and painful in six (13.3%) patients. Inclination was without limitations in 36 (78.3%), with slight limitations in four (8.6%), and painful in six (13.0%) patients.

According to Odom´s criteria, 35 (76.1%) patients reported clinical success. A compilation of EQ-5D questionnaire results is shown in Table 4.

**Table 4:** EQ-5D questionnaire and results.

Dimension	Question	Results
**Mobility**		
Level 1	I have no problems in walking about	57.9%
Level 2	I have some problems in walking about	42.1%
Level 3	I am confined to bed	0%
**Self-Care**		
Level 1	I have no problems with self-care	92.1%
Level 2	I have some problems washing or dressing myself	7.9%
Level 3	I am unable to wash or dress myself	0%
**Usual Activities (e.g. work, study, housework, family or leisure activities)**		
Level 1	I have no problems with performing my usual activities	68.4%
Level 2	I have some problems with performing my usual activities	31.6%
Level 3	I am unable to perform my usual activities	0%
**Pain/Discomfort**		
Level 1	I have no pain or discomfort	47.4%
Level 2	I have moderate pain or discomfort	53.6%
Level 3	I have extreme pain or discomfort	0%
**Anxiety/Depression**		
Level 1	I am not anxious or depressed	71.1%
Level 2	I am moderately anxious or depressed	18.4%
Level 3	I am extremely anxious or depressed	10.5%

### Repeated cervical spine procedure

Eight patients underwent repeated cervical procedure (17.4%) with a mean duration from CDA procedure to repeated procedure of 83 months (range 7–165 month). A removal of the CDA was performed in three (6.5%) patients. Four patients developed sASD (8.6%) and underwent repeated procedure. A detailed compilation of all repeated procedures with respect to underlying diagnosis, diseased segment and surgical technique is shown on Table 5.

**Table 5:** Repeated procedures.

Segment(s) of initial CDA procedure	Diagnosis at repeated procedure	Location of diseased segment	Number of patients	Surgical technique at repeated procedure(s)	Duration form initial CDA to repeated procedure (month)
C5/6	Radiculopathy caused by foraminal stenosis	Index	1	CDA removal Decompression and fusion of C5/6 via ACDF + CP	105
C5/6	Caudal sASD due to stenosis	C6/7	1	CDA removal Decompression and fusion of C5/6 and C6/7 via ACDF + CP	130
C5/6*	Loosening of prosthesis	Index	1*	CDA removal Decompression and fusion C5/6 via ACDF + CP	7
C5/6	Cranial and caudal sASD due to stenosis	C4/5 C6/7	1	Decompression and fusion of C4/5 and C6/7 via ACDF	70
C4/5 & C5/6	Caudal sASD radiculopathy due to calcified lateral disc herniation and foraminal stenosis	C6/7	1	Posterior foraminotomy C6/7	124
C5/6	Cranial and caudal sASD and index segment radiculopathy due to osseous foraminal stenosis	C4/5 C5/6 C6/7	1	Posterior foraminotomy at C4/5, C5/6 and C6/7	165
C6/7	Cervical disc herniation	C4/5	1	CDA at C4/5	21
C3/4	Cervical disc herniation	C5/6	1	Decompression and fusion of C5/6 via ACDF	45

*Patient developed sASD at C4/5 and underwent ACDF + CP 6 years after CDA removal.

### MRI evaluation and statistical comparison

Preoperatively, the mean SDI of the cervical spine was mild in 30.3% of segments, moderate in 64.2% of segments, and severe in 5.5% of segments. Signs of spondylosis and facet joint arthrosis were noted in 52.2% of the index segments, in 50% of the cranial adjacent segments, and in 52.2% of the caudal adjacent segments.

The mean SDI of cranial adjoining segments was 0.423, the mean SDI of adjacent segments was 0.462, and the mean SDI of caudal adjoining segments was 0.371. A normal distribution of SDI values was noted and there was no significant difference between preoperative and the final follow-up SDI for each of the assessed segments. The percentage of mild SDI, moderate SDI, and severe SDI of the proximal and distal cranial adjoining segments, the cranial and caudal adjacent segments, and the proximal and distal caudal adjoining segments at preoperative and final follow-up time points are shown on Table 5. The mean preoperative and final follow-up SDI of each segment was compared to identify degenerative changes over time. A detailed compilation of mean preoperative and follow-up SDI scores and the p-value for comparison is shown on Table 6. The reviewers had agreement in 78.9% for SDI. The intra class correlation (ICC) was 0.876 which is considered very good reliability.

**Table 6:** Comparison of SDI.

	Preoperative	Follow-up	*P* value
Distal cranial adjoining	Mean SDI 0.304	Mean SDI 0.429	0.329
*Mild SDI*	*37.5%*	*57.7%*	
*Moderate SDI*	*62.5%*	*42.3%*	
*Severe SDI*	*0%*	*0%*	
Proximal cranial adjoining	Mean SDI 0.427	Mean SDI 0.428	0.983
*Mild SDI*	*30.0%*	*20.0%*	
*Moderate SDI*	*70.0%*	*71.4%*	
*Severe SDI*	*0%*	*8.6%*	
Cranial adjacent	Mean SDI 0.524	Mean SDI 0.517	0.830
*Mild SDI*	*9.5%*	*5.6%*	
*Moderate SDI*	*71.4%*	*77.8%*	
*Severe SDI*	*19.0%*	*16.7%*	
Caudal adjacent	Mean SDI 0.376	Mean SDI 0.421	0.207
*Mild SDI*	*23.8%*	*19.4%*	
*Moderate SDI*	*71.4%*	*80.6%*	
*Severe SDI*	*4.8%*	*0%*	
Proximal caudal adjacent	Mean SDI 0.296	Mean SDI 0.334	0.322
*Mild SDI*	*47.6%*	*41.7%*	
*Moderate SDI*	*47.6%*	*55.6%*	
*Severe SDI*	*4.8%*	*2.8%*	
Distal caudal adjacent	Mean SDI 0.387	Mean SDI 0.285	0.331
*Mild SDI*	*47.6%*	*44.4%*	
*Moderate SDI*	*52.4%*	*55.6%*	
*Severe SDI*	*0%*	*0%*	

The univariate linear regression analysis revealed that the type of prosthesis and gender had no statistically significant influence on the SDI on the adjacent and the adjoining segments.

The number of operated segments had a statistically significant influence on the SDI at the proximal cranial adjoining segment (*p* value: 0.037). The years of follow-up had a statistically significant influence on the SDI at the proximal cranial adjoining segment (*p* value: 0.020). No statistically significant influences were noted for these parameters on the other assessed segment.

## Discussion

ACDF is a standardized procedure that has shown to achieve high rates of clinical success in long-term follow-up studies^6,7,21^. The factor that ACDF might accelerate the degenerative process of adjacent segments is often neglected by surgeons. The goals of anterior cervical spine surgery for degenerative disorders, in general, are decompression of the spinal cord and nerve roots, restoration of intervertebral height, and restoration of lordosis. CDA was developed to maintain the motion of the segment after decompression. The reduction of ASD was seen as a potential advantageous side effect but not the primary intention of the developers.

Over the past decade, several prospective randomized trials were performed to assess clinical and radiographical outcome as well as reoperation rates following CDA procedures. Even though there are some reports that CDA is superior to ACDF for clinical outcome and radiographic findings, the short- to mid-term data is inconclusive on the superiority of CDA over ACDF concerning ASD and repeated procedure for sASD^16,22–25^.

Badhiwala et al. performed a meta-analysis of prospective randomized trials and reported that the rate of repeat procedure for sASD was 2.3% for one-level procedures and 1.7% for two-level procedures at 2 years. This rates increased to 4.3% for one-level and 5.1% for two-level procedures at 7 years of follow-up. Reported rates for repeat procedure at the index level varied from 2.8 to 3.2% at 2 years and 4.2% to 5.2% at 7 years of follow-up^26^.

In contrast to the aforementioned meta-analysis, the overall rate of repeated procedure in the present study was 17.4% and therefore considerably higher. There might be some factors that contribute to this finding. A closer look at the data reveals that the rate for sASD within 6 years of follow-up was only 2.1% and therefore similar to the rates reported by Badhiwala et al. After more than 6 years of follow-up the rate for repeat procedure due to sASD increased to 8.9% which is similar to the rate of 9.7% in a 10-year follow-up study reported by Lavelle et al.^15^ Also, the repeated procedure rate at the index level in this study (i.e. 4.3%) was similar to the rates reported by Badhiwala et al. In addition to procedures at the index segment and adjacent segments a total of 4.3% of patients underwent surgery at the adjoining segments which contribute to a higher overall repeated procedure rate.

The patient selection differs between prospective randomized trials and the herein presented patient cohort. Randomized trials usually adhere to strict inclusion criteria such as single-level pathology without substantial degeneration or other pathologies at the adjacent segments. In the present study, the main indication (91.3%) for CDA procedure was cervical radiculopathy due to disc herniation. The mean age of patients for CDA procedure was 45 years and therefore comparable to other studies^27^. However, the preoperative SDI of the adjacent segments was moderate in about 70% of patients. This reveals that degenerative findings at the adjacent segment were present preoperatively but were not considered to be severe enough to exclude the patient as a candidate for CDA procedure.

However, even though patients of the present study did not undergo a rigorous selection process as in other prospective randomized studies the NDI of 12% and clinical success of 76% were also similar compared to results from long-term follow-up prospective randomized study^15^.

The sagittal and the segmental alignment of the cervical spine might have been an influence on the clinical outcome. It would have been interesting to see if cervical lordosis might change over time. Unfortunately, the design of the present study did not allow performing a radiograph at the final follow-up. Therefore, no profound conclusion can be made on this topic. In the author's opinion assessment of the segmental angle of the diseased segment via MRI is not ideal due to the supine positioning of the patient and the movement of the segment.

However, the MRI analysis of the present study demonstrated that adjacent, as well as adjoining segment, demonstrated mild to moderate degeneration before CDA procedure. A multitude of studies analyzed different prostheses and its clinical outcome. Clinical outcome was comparable for different types of prosthesis^28^. However, biomechanical characteristics and their influence on clinical outcome have not been assessed thoroughly so far. Muhlbauer et al. recommended that only prostheses with flexible biomechanical properties should be used in clinical practice^29^. The MRI assessment showed that progressive degeneration most often occurred at the cranial adjacent segment. This finding is not really surprising, because CDA was performed in about 40% at the segment C6/7, and it is well known that the segments caudal to C6/7 have other biomechanical characteristics and less motion in general due to the rigid thorax.

However, the statistical analysis of the MRI data did not reveal the relevant deterioration of adjacent segment degeneration based on our 5-step grading system. This is in contrast to the long-term study performed by Genitiempo et al. who assessed the grade of segment degeneration using the Pfirrmann grading system^30^. Two reasons might add to this different finding. First, Genitiempo et al. included patients who had a mean age of 42 years at a mean follow-up of 18 years^30^. The age in this cohort was considerably lower compared to the mean age of 56 in the present study. The natural history of degeneration might have influenced this MRI-based finding. Furthermore, patients were operated on for soft disc herniation only, which occurs in an early stage of disc degeneration.

Also, it should be stated that the mobility of the CDA prosthesis might contribute to degeneration as well. An 80% range of motion rate for CDA has been reported 10 years after implantation^27,31^ with a decrease to 56% after 18 years of follow-up^32^. In theory, loss of mobility should increase the stress on the adjacent segment which then could accelerate degeneration as well. The number of studies that assessed the CDA mobility and MRI-based degeneration more than 15 years after surgery is limited. In the present study, the mean follow-up length was 11 years, therefore we can assume that the mobility rate of our cohort was higher compared to the 18 years follow-up study of Genitiempo^30^, which could have been contributed to the none statistically significant changes in degeneration.

The MRI-based results of this study can be compared to other long-term follow-up studies which have assessed the grade of degeneration of cervical spine segments following ACDF and ACDF + CP procedures. At a mean follow-up of 25 years following an anterior fusion procedure about 80% of all cranial and about 60% of all caudal adjacent segments showed moderate and severe degeneration according to the SDI^18,20^. The MRI findings of the present study showed that moderate and severe SDI scores for cranial and caudal segments were found in over 80% of patients as well. In a previous study reoperation and clinical success rate of patients who underwent ACDF or ACDF + CP with a mean follow-up of 25 years were both not inferior to the rate of our CDA cohort. In both cohorts` patients did not undergo a rigorous selection process as contrarily done in prospective randomized trials. In the authors´ opinion, it is therefore not clear if the non-significant change of degeneration of the adjacent segment according to the SDI on MRI has an influence on the overall clinical success rate in the long-term follow-up. The results of the present study were similar in most aspects compared to prospective randomized trials even though the selection process of patients was not as strict as in those trials.

## Conclusion

Within 11-year follow-up after CDA, the rate of clinical success was 76%, the overall reoperation rate was 17%, including 9% reoperation rate owing to sASD. Follow-up MRIs did not confirm hastened segmental degeneration following CDA.

## Data Availability

All data generated or analysed during this study are included in this published article.
